# Florid cemento-osseous dysplasia: a contraindication to orthodontic treatment in compromised areas

**DOI:** 10.1590/2177-6709.23.3.026-034.oin

**Published:** 2018

**Authors:** Alberto Consolaro, Sergio Rafael Baggio Paschoal, Jose Burgos Ponce, Dario A. Oliveira Miranda

**Affiliations:** 1 Universidade de São Paulo, Faculdade de Odontologia de Bauru (Bauru/SP, Brazil).; 2 Universidade de São Paulo, Faculdade de Odontologia de Ribeirão Preto, Programa de Pós-graduação em Odontopediatria (Ribeirão Preto/SP, Brazil).; 3 Private clinic (Ribeirão Claro/PR, Brazil).; 4 Centro Universitário de Adamantina, Curso de Medicina, Disciplina de Patologia (Adamantina/SP, Brazil).; 5 Universidade Estadual de Feira de Santana, Curso de Odontologia (Feira de Santana/BA, Brazil).

**Keywords:** Bone dysplasia, Florid cemento-osseous dysplasia, Osteomyelitis, Bone diseases

## Abstract

Florid cemento-osseous dysplasia is a sclerosing disease that affects the mandible, especially the alveolar process, and that is, in most cases, bilateral; however, in some cases it affects up to three or even four quadrants. During the disease, normal bone is replaced with a thinly formed, irregularly distributed tissue peppered with radiolucent areas of soft tissue. Newly formed bone does not seem to invade periodontal space, but, in several images, it is confused with the roots, without, however, compromising pulp vitality or tooth position in the dental arch. There is no replacement resorption, not even when the images suggest dentoalveolar ankylosis. Orthodontists should make an accurate diagnosis when planning treatments, as this disease, when fully established, is one of the extremely rare situations in which orthodontic treatment is contraindicated. This contraindication is due to: (a) procedures such as the installment of mini-implants and mini-plaques, surgical maneuvers to apply traction to unerupted teeth and extractions should be avoided to prevent contamination of the affected bone with bacteria from the oral microbiota; and (b) tooth movement in the areas affected is practically impossible because of bone disorganization in the alveolar process, characterized by high bone density and the resulting cotton-wool appearance. Densely mineralized and disorganized bone is unable to remodel or develop in an organized way in the periodontal ligaments and the alveolar process. Organized bone remodeling is a fundamental phenomenon for tooth movement.

## RESUMO

A displasia cemento-óssea florida é uma doença óssea esclerosante exclusiva dos maxilares, relacionada ao osso do processo alveolar e, na maioria dos casos, envolvendo bilateralmente a mandíbula; mas há casos em que envolve três ou até os quatro quadrantes. Nesse processo, troca-se o osso normal por um tecido densamente formado, irregularmente distribuído e salpicado por áreas radiolúcidas com tecido mole. O osso neoformado parece não invadir o espaço periodontal, mas, em muitas imagens, confunde-se com as raízes, sem comprometer a vitalidade pulpar e a posição dentária na arcada. Não há reabsorção dentária por substituição, mesmo quando as imagens sugerem anquilose alveolodentária. Um diagnóstico preciso por parte do ortodontista deve ser feito em seus planejamentos, visto que essa doença, quando se encontra plenamente instalada, representa uma das raríssimas situações em que o tratamento ortodôntico está contraindicado. Nesses casos, o tratamento ortodôntico está contraindicado porque: a) alguns procedimentos, como a aplicação de mini-implantes e miniplacas, manobras cirúrgicas para tracionamento de dentes não irrompidos e exodontias, devem ser evitados, para se impedir a entrada de bactérias da microbiota bucal no osso comprometido; e b) a possibilidade de movimentação dos dentes nas áreas comprometidas praticamente inexiste, pela desorganização óssea no processo alveolar, caracterizada por elevada densidade óssea, que gera as imagens tipo flocos de algodão. O osso densamente mineralizado e desorganizado não é capaz de se remodelar e desenvolver organizadamente, nos ligamentos periodontais e no osso do processo alveolar. A remodelação óssea organizada é fundamental como um dos fenômenos necessários para o deslocamento dos dentes.

Orthodontic movement requires the jawbones to be actively remodeling, so that the forces applied to the teeth reorganize and, at the same time, change or reformat the design of jawbones, to restore the patient’s adequate and planned function and esthetics. Without a normal adequate bone remodeling process, there is no orthodontic movement, or, when there is some movement, it is not enough to restore the normal esthetics and functions.

In this new century, Brazil has adopted a new National Oral Health Plan, and at the same time, social and economic conditions allowed lower-income populations to have access to health services. Over 75% of the Brazilian population has genes of African ethnicity groups. At the same time, there has been an increase in the knowledge about florid cemento-osseous dysplasia and the number of diagnosed cases, particularly by using panoramic radiographs. 

**Figure 1:**
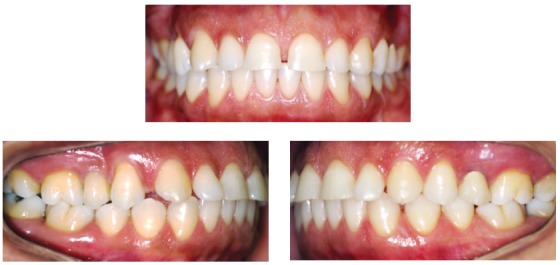
Case of florid cemento-osseous dysplasia, seen for evaluation of possible orthodontic treatment. Black woman aged 45 years and 3 months. No symptoms associated with lesion and no systemic diseases reported. Frontal and lateral views.

**Figure 2:**
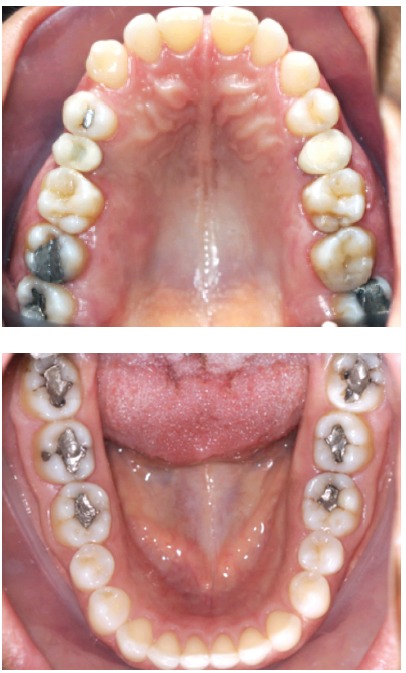
Same case of florid cemento-osseous dysplasia as in Figure 1, seen for evaluation of possible orthodontic treatment; occlusal view. No clinical signs of disease.

**Figure 3:**
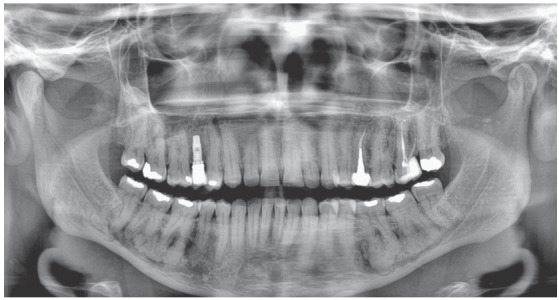
Panoramic radiograph of same clinical case with florid cemento-osseous dysplasia seen for evaluation of possible orthodontic treatment. Mandibular lesions are mixed, some radiopaque, some radiolucent, and are confused with roots of molar teeth, as they are randomly distributed in posterior region. Lesions have a cotton-wool appearance (or “flower beds”). Maxilla was not affected; implant is normal and functioning.

**Figure 4:**
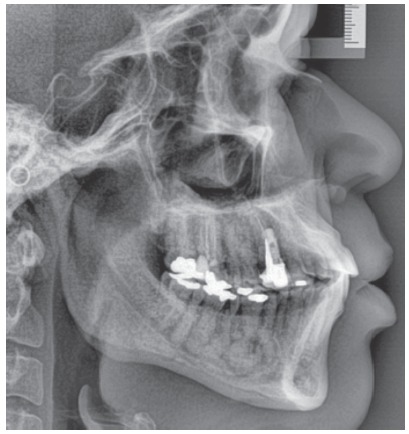
Other radiographic findings of same clinical case with florid cemento-osseous dysplasia seen for evaluation of possible orthodontic treatment. Mandibular lesions are mixed and overlap roots of molar teeth, as they are randomly distributed in posterior region; cotton-wool appearance.

Florid cemento-osseous dysplasia is not rare, and a large number of cases are diagnosed in the imaging documentation centers (Figs 1 to 4). It is more frequent in black women about 40 years old at the time of diagnosis, with a prevalence of 5.5% among them.^1^ ^-^ ^5^ Although common among women, it is not exclusive of this sex, and it may also affect white people. 

Florid cemento-osseous dysplasia, a sclerosing disease that affects the mandible, is associated with the alveolar process and, in most cases, is bilateral; however, it may affect up to three or even four quadrants in some cases. In this process of intense and abnormal bone sclerosis, normal bone is replaced with a densely formed tissue, irregularly distributed and peppered with radiolucent areas of soft tissue found primarily in the alveolar process (Figs 1 to 4), including the interdental and interradicular septa. After some time, newly formed bone invades the periodontal space and is confused with the dental roots, without, however, compromising pulp vitality and tooth position in the dental arch.

The characteristics described above indicate that orthodontists must make an accurate diagnosis of florid cemento-osseous dysplasia in the analyses conducted for their treatment planning, as this disease, when fully established, is one of the rare conditions that contraindicate orthodontic treatment.

## BONE AND ITS CHARACTERISTICS: BASES TO UNDERSTAND THE CLINICAL AND BIOLOGICAL IMPORTANCE OF FLORID CEMENTO-OSSEOUS DYSPLASIA.

The human skeleton undergoes full renewal every 1 to 4 years in children and 4 to 10 years in adults. Bones have both soft and mineralized tissues. Osteoblasts, osteocytes and osteoclasts are mature bone cells that, in association with other components, such as macrophages, promote bone remodeling, which exists, primarily, to control the levels of mineral ions in the blood and tissues. 

Constant bone remodeling gives bone greater adaptability. According to the stimuli applied to its structure, the form and volume that define bone design may be remodeled. This adaptive and reactional capacity of bone is clinically very important in everyday practice in Dentistry. 

The reactional capacity of bone and its resistance to stimuli or aggressors depend on three fundamental factors, which may determine the type of lesion a certain cause may inflict on the affected bone: 

**1) Local bone morphology**

A more compact or dense cancellous bone has small medullary spaces, and, consequently, not much space for more abundant inflammatory exudates. Any inflammation may increase, at an early stage, the pressure inside these reduced medullary spaces, thus compressing the vases and creating barriers to venous return, which leads to medullary tissue necrosis in a shorter time. An area of necrotized medullary tissue may be the ideal site for bacteria to lodge and build microbial biofilm.

On the other hand, bone with more space or loosely distributed trabeculae offers more space for inflammatory exudates and infiltrate when there is an insult.

Logical deduction leads to the conclusion that bone that is more compact is much stronger physically, but fragile biologically, as it requires that the inflammatory process function more rapidly. The opposite is seen when the bone is less compact but spongier: inflammation tools have more time and room to act against aggressors.

**2) Insult intensity and duration**

In the same way as other insults, mild and constant irritation, or irritation described as chronic, leads to initial acute inflammation, but rapidly goes into a mild or moderate chronic phase, in which there is restricted accumulation of mediators at the site of inflammation. 

Several inflammatory exudate mediators induce bone resorption, but some have bipolar effects: when concentration is high, they induce a predominantly clastic activity; but when levels are low in the same bone environment, they induce synthesis and osteoblastic action, with bone formation predominantly developing on trabecular and cortical surfaces.

Inducing mediators of new bone formation on the trabecular and subperiosteal surfaces gradually change local bone morphology, which remains organized. As irritation increases its insulting power, the reaction to form new bone may still occur, but in a not so organized way.

Rapid and intense irritation, or that referred to as acute, in the same way as all other insults, promotes acute initial inflammation, but much more exudative and rich in terms of mediators inducing bone resorption, and may, therefore, induce the formation of areas of medullary and endosteal tissue and osteocyte necrosis.

Mild or chronic insults induce new bone formation or production, predominantly as synthesis, whereas severe or acute insults lead to bone resorptive, osteolytic or destructive reactions.

**3) Host systemic state.**

The systemic state of the host is determinant in bone reactions to insults. Osteomyelitis occur only in:

1) Patients systemically compromised, such as those with uncontrolled diabetes, immunodepression and anemias, oncologic patients, patients undergoing chemotherapy for cancer, chronic alcoholism and undernourished patients with irregular diets or socioeconomic difficulties.
2) Patients with localized sclerosing bone diseases, such as florid cemento-osseous dysplasia and Paget’s disease of bone.

In radiotherapy, osteoradionecrosis and osteoradiomyelites occur when common bacteria invade the areas of irradiated bone, as a result of: a) chronic hypoxia promoted by endarteritis obliterans, which partially blocks blood passage to the cells; b) hypovascularization; c) hypocellularity in the irradiated area because of the very small mitotic index, which greatly reduces reparative and reactional capabilities; and d) death of osteocytes, cells that have a fundamental role in bone histophysiology. 

When the patient is systemically healthy, the same causes that induced osteomyelitis promote osteitis, also an inflammation, but local and focal and with less serious consequences, because the osteolytic areas are restricted and small, the areas of bone sclerosis are predominant, and symptoms are very few. The prognosis of osteitis is very good.

## THE ALSO CALLED ODONTOGENIC FIBRO-OSSEOUS LESIONS

Since 1971, the WHO, in studies such as the one conducted by Pindborg and Kramer,^6^ among others, has attempted to unify the nomenclature and classification of odontogenic neoplasia, among which are the fibro-osseous lesions. In that first study, six lesions were defined, four of which were classified in a group called cementomas: cementing fibroma, periapical cemental dysplasia, benign cementoblastoma and gigantiform cementoma. The other two diseases included as odontogenic fibro-osseous lesions were ossifying fibroma and fibrous dysplasia, although much more closely associated with bone.

Defining a uniform nomenclature and classification of these lesions is difficult because it is impossible to distinguish whether the mineralized material in these lesions is cement or bone, even when analyzed under transmission or scanning electron microscopy or immunocytochemistry. In 1989, Burkhardt^7^ analyzed two fibro-osseous lesions using optical and electron microscopy and immunocytochemistry and found that some mineralized tissues may also have a dentinal nature.

In the present study, the priority and focus is on one of these formerly called cementomas, which was also called a gigantiform cementoma for a long time. Currently, the classification most commonly used universally is florid cemento-osseous dysplasia. 

## IS THE CIGANTIFORM CEMENTOMA THE SAME AS FLORID CEMENTO-OSSEOUS DYSPLASIA?

Initially, gigantiform cementoblastomas were characterized as a typically multiple and symmetrically distributed benign odontogenic lesion that affected middle-aged black women. Radiographically, they are dense lesions and lobulated masses that are microscopically composed of tissue that resembles cement. 

Later, in 1976, the term florid cemento-osseous dysplasia was coined by Melrose et al^8^ to describe an exuberant multi-quadrant fibro-osseous lesion in which the bone was gradually replaced with fibro-cement tissue, as described for gigantiform cementomas. 

The name florid cemento-osseous dysplasia was widely accepted to describe also the condition previously described as multiple cemento-ossifying fibroma, multiple sclerosing osteomyelitis and sclerotic cemental masses. The term florid cemento-osseous dysplasia was also adopted for gigantiform cementomas. 

However, the need to use the term gigantiform cementoma or familial gigantiform cementoma was demonstrated by Young et al^9^ in 1989, who described five generations of a family with familial gigantiform cementoma of dominant autosomal character. Although the two lesions are indistinguishable under imaging and microscopic examination, familial gigantiform cementomas have a familial or hereditary history, whereas florid cemento-osseous dysplasia does not. 

Florid cemento-osseous dysplasia, but not gigantiform cementomas, has a greater prevalence among black women. Familial gigantiform cementoma is characterized by great expansive and asymmetrical growth, not found in florid cemento-osseous dysplasia. Several authors recommend that the two lesions, although they have some points in common, should be classified separately as two distinct entities.

## FLORID CEMENTO-OSSEOUS DYSPLASIA MAY BE FOCAL OR GENERALIZED

The term florid cemento-osseous dysplasia was used for the first time in 1976, when Melrose et al^8^ described an exuberant multi-quadrant fibro-osseous lesion characterized by bone replacement with fibro-cement in the jawbones. That classification was largely accepted to describe conditions that had been previously reported as multiple cemento-ossifying fibroma, sclerosing osteomyelitis and sclerotic cemental masses. 

Summerlin and Tomich^10^ reanalyzed cases of more localized lesions, such as cementifying fibroma and central ossifying fibrous dysplasia of the jawbones, chronic sclerosing osteomyelitis, sclerotic cemental masses and fibro-osseous lesions, under several focuses, in 1994. They concluded that the clinical entity called focal cemento-osseous dysplasia was an initial phase of florid cemento-osseous dysplasia that gradually compromises the other areas to result in the complete or generalized symptoms of the disease. This initial lesion of florid cemento-osseous dysplasia, called focal cemento-osseous dysplasia, may simulate several other fibro-osseous lesions of the jawbones, especially cementifying or central ossifying fibromas, and special attention should be paid at the time of differential diagnosis, as its nature is benign, with variable degrees of local aggressiveness. Focal cemento-osseous dysplasia seems to compose only a localized and still initial form of florid cemento-osseous dysplasia, but it is not a new clinical entity or disease.

In contrast, periapical cemental or cemento-osseous dysplasia in the region of the mandibular incisors may only be a uniform manifestation of the same focal cemento-osseous dysplasia, but this is still controversial. May periapical cemental or cemento-osseous dysplasia evolve into florid cemento-osseous dysplasia? Clinical experience and literature reveal that it may not. Their occurrence together in the same patient seems to be a fortuitous and rare occurrence.

## HOW DOES FLORID CEMENTO-OSSEOUS DYSPLASIA INITIATE?

In a certain point of the region of the mandibular molars and premolars, probably simultaneously in both sides, bone tissue is resorbed and replaced with fusiform and polyhedral cells that form discretely fibrosed connective tissue. These areas, if examined using imaging techniques, are radiolucent or hypodense, as they are osteolytic and irregular. 

After some time, these polyhedral cells initiate the deposition of an irregularly and randomly distributed, disorganized collagen matrix, over which basophilic mineralized bone begins to form. Its structure and organization resemble that of immature or still disorganized mature bone, or, at other times, that of a cementum-like tissue. 

The radiolucent areas reveal irregular and focal irregular radiopaque areas that gradually unite to form radiopaque masses, usually surrounded by irregular radiolucent areas that interface with normal neighboring bone and are filled with soft tissue that has not yet been mineralized. In these initial stages, there are no clinical signs, not even any signs on CT scans or radiographs (Figs 1 to 4). This process tends to affect the mandible bilaterally and asymptomatically and gradually increases until it is eventually diagnosed on imaging studies indicated due to other clinical conditions.

Which factor triggers this pathologic process? Florid cemento-osseous dysplasia probably originates in the periodontal ligament as a result of irregular cementum formation and a disorder in the differentiation of periodontal ligament stem cells. Under normal conditions, these cells differentiate into cementoblasts, fibroblasts and osteoblasts of the alveolar bone every day.

This disorder of the periodontal ligament stem cells may explain why this mineralized material has already been described as similar to cementum and bone, between fibrous tissue similar to the periodontal ligament. It may also explain why these mineralized masses are not encapsulated.

## CLINICAL AND IMAGING FEATURES OF FULLY ESTABLISHED FLORID CEMENTO-OSSEOUS DYSPLASIA

Florid cemento-osseous dysplasia has no symptoms or clinical signs. Teeth in the areas affected by this condition show pulp vitality and normal color. There is no expansion of the cortical bone, and, therefore, no jawbone volume increase or asymmetry. The areas affected have a normal texture to palpation, and the overlying mucosa is completely normal (Figs 1 to 4).

Radiographically, it is characterized by radiopaque masses with a cotton-wool appearance (irregular “flower beds”), especially in the periapical region. Imaging studies reveal mixed radiopaque and radiolucent areas (Figs 1 to 4).

In the space limited by the jawbone cortical bones, florid cemento-osseous dysplasia gradually involves all alveolar process in the interdental and interradicular spaces. In the region of the affected teeth, it is not possible to detect the radiolucent periodontal space or the radiopaque lamina dura, but there is no root replacement reabsorption, no matter how advanced the disease is, which is a sign of presence of the periodontal ligament.

Around the so-called “cotton wads”, which are the radiopaque masses of cementum-like or osteoid tissue, there are radiopaque spaces filled with soft tissue not yet mineralized (Figs 1 to 4). This overlapping reveals that these radiopaque and radiolucent masses are random in a variety of radiographic images.

The aspects described above are particularly detailed on CT scans and three-dimensional reconstructions. Between these masses and teeth, no periodontal spaces are seen at some points, not even in thinner CT sections, but there is no replacement resorption. CT sections reveal that these masses extend from one cortical to the other.

With time, the involvement of the jawbone, particularly the mandible, may extend from the area of third molars to that of canines, and lesions may be found in both quadrants. In some cases, these radiopaque masses surround and involve unerupted teeth that may be in the region.

In most cases, only two mandibular quadrants are affected; but in some other cases, the posterior regions of the bone are also affected, often less severely than the other areas.

A very important clinical sign for the diagnosis of florid cemento-osseous dysplasia is the appearance of a yellowish material, similar to bone, that comes out of the buccal mucosa through perforations that are unexplainable at first. This may indicate contamination of a low degree of virulence in the lesion, which has not yet been diagnosed at the time, and of which the patient is still unaware.^11^ ^,^ ^12^

## HOW SHOULD CASES OF FLORID CEMENTO-OSSEOUS DYSPLASIA BE MANAGED CLINICALLY?

The reactional and reparative capacity of bone with florid cemento-osseous dysplasia is severely limited. There are few and very small medullary spaces. Any amount of inflammatory exudate (liquid) and infiltrate (cells) compresses the vases at an early stage, which leads to necrosis. 

Any common bacteria of the buccal microbiota, if they reach the sclerotic bone affected by florid cemento-osseous dysplasia, promotes inflammation that rapidly progresses to chronic purulent or suppurative osteomyelitis, generating multiple fistulas and mutilating consequences for the patient.^1^ ^,^ ^2^ ^,^ ^13^ ^-^ ^15^

These bacteria rapidly form a mixed microbiota that colonizes the sclerotic bone surfaces and hide in the necrotic areas, which complicates the access and action of drugs. In this case, treatment should be surgical, with mandibular debridement and significant loss of mandibular structure, as in the case of primary chronic suppurative osteomyelitis.

The main treatment strategy for florid cemento-osseous dysplasia is to avoid the contact of the affected bone with the oral microbiota, which happens in cases of chronic inflammatory periodontal disease associated with dental bacterial plaque, caries followed by pulp necrosis and chronic periapical diseases, tooth and jawbone trauma, surgery with extractions and placement of implants. Endodontic treatment should only be made when loss of tooth vitality is confirmed, and should be conducted under preventive antibiotic therapy after a clear diagnosis of florid cemento-osseous dysplasia has been made.^16^

The oral mucosa should not be ulcerated or operated on, and no punch biopsy should be obtained to make a microscopic diagnosis of florid cemento-osseous dysplasia. The final diagnosis of florid cemento-osseous dysplasia should be made according to clinical and imaging data only.

Simple periodontal scaling, implant placement or extraction is sufficient to induce chronic suppurative osteomyelitis secondary to florid cemento-osseous dysplasia^13^. When urgency or emergency procedures, as well as other procedures classified as unavoidable, expose the affected jawbone to the oral environment, strict antibiotic therapy, in terms of duration, dose and antibiotic choice, should be prescribed.

Despite all measures taken, the patient with florid cemento-osseous dysplasia may, with time, develop suppurative osteomyelitis secondary to florid cemento-osseous dysplasia after contamination of the bone by bacteria from transient bacteremia that the human body has practically on a daily basis. Transient bacteremia may occur during personal hygiene procedures, mastication or other very simple daily actions.

Although well described in the literature, several dentists still find it difficult to diagnose florid cemento-osseous dysplasia.^3^ ^,^ ^4^ ^,^ ^17^ ^,^ ^18^ Sometimes when there is no clear diagnosis, the clinical signs and symptoms of florid cemento-osseous dysplasia are assigned to the action of bisphosphonates on the process of bone remodeling, which is a serious conceptual and practical mistake.^19^

Patients and their families should be aware of their condition after a solid diagnosis of florid cemento-osseous dysplasia has been made, to avoid equivocal treatment options made by other healthcare professionals and to take all the necessary steps to avoid the access of bacteria to the affected area, by adopting a healthy and focused life style.

## WHAT ARE THE DENTAL TREATMENT CONTRAINDICATIONS IN CASES OF FLORID CEMENTO-OSSEOUS DYSPLASIA?

Surgical procedures should be avoided to ensure that bacteria from the microbiota do not reach the bone and, consequently, lead to chronic suppurative osteomyelitis secondary to florid cemento-osseous dysplasia.

Extractions should be made only when extremely necessary. Whenever possible, extractions of unerupted teeth should be avoided, and these teeth should be followed up radiographically every year.

Osseointegrated implant placement in the affected area is completely contraindicated, as the risk to develop secondary chronic suppurative osteomyelitis is very high.

Surgical procedures of any nature should be avoided, including those for orthodontic treatments, such as traction and the placement of mini-implants and mini-plaques. 

Orthodontic treatment is contraindicated because the procedures mentioned above should be avoided; moreover, the teeth in the affected areas should not be moved. Densely mineralized and disorganized bone cannot remodel and develop on the periodontal ligament and alveolar bone, and these are the phenomena required for tooth movement. In other words, teeth are not capable of moving in areas with florid cemento-osseous dysplasia. In some microscopic sections of disease samples, there is not a single visible full periodontal ligament, the structure responsible for orthodontic movement.

Some exceptions to the contraindications for procedures in the jawbones of patients with florid cemento-osseous dysplasia should be made clear. If at a certain time the disease affects only the mandible, implant placement and tooth movement in the areas not affected and in the maxilla may be performed. The same applies to endodontic treatments and extractions, even of unerupted teeth. The placement of mini-implants and mini-plaques should be restricted because of bacterial contamination while they are in the mouth, which may result in daily bacteremia.

## FINAL CONSIDERATIONS

Orthodontists should make an accurate diagnosis when planning treatments, as florid cemento-osseous dysplasia, when fully established, is one of the extremely rare situations in which orthodontic treatment is contraindicated. The reasons for this contraindication are: 

1) Some procedures, such as the installment of mini-implants and mini-plaques, surgical maneuvers to apply traction to unerupted teeth and extractions, should be avoided to prevent contamination with bacteria from the oral microbiota in the bone affected.

2) Tooth movement in the areas affected is practically impossible because of bone disorganization in the alveolar process, characterized by a high bone density and the resulting cotton-wool appearance. Densely mineralized and disorganized bone is unable to remodel and develop in an organized way in the periodontal ligaments and in the alveolar bone. Organized bone remodeling is a fundamental phenomenon for the success of orthodontic movement.

## References

[1] Das BK, Das SN, Gupta A, Nayak S (2013). Florid cemento-osseous dysplasia. J Oral Maxillofac Pathol.

[2] El-Naggar AK, Chan JKC, Grandis JR, Takata T, Slootweg PJ (2017). WHO classification of head and neck tumors.

[3] Alsufyani NA, Lam EW (2011). Osseous (cemento-osseous) dysplasia of the jaws clinical and radiographic analysis. J Can Dent Assoc.

[4] Na A, Ewn L (2011). Cemento-osseous dysplasia of the jaw bones key radiographic features. Dentomaxillofac Radiol.

[5] Senia ES, Sarao MS (2015). Periapical cemento-osseous dysplasia a case report with twelve-year follow-up and review of literature. Int Endod J.

[6] Pindborg JJ, Kramer IRH (1971). Histological typing of odontogenic tumours, jaw cysts and allied lesions.

[7] Burkhardt A (1989). Dentin formation in so-called fibro-osteo-cemental lesions of the jaw histologic, electron microscopic and immunohistochemical investigations. Oral Surg Oral Med Oral Pathol.

[8] Melrose RJ, Abrams AM, Mills BG (1976). Florid osseous dysplasia a clinical-pathological study of thirty-four cases. Oral Surg Oral Med Oral Pathol.

[9] Young SK, Markowitz NR, Sullivan S, Seale TW, Hirschi R (1989). Familial gigantiform cementoma classification and presentation of a large pedigree. Oral Surg Oral Med Oral Pathol.

[10] Summerlin DJ, Tomich CE (1994). Focal cemento-osseous dysplasia a clinicopathologic study of 221 cases. Oral Surg Oral Med Oral Pathol.

[11] Glascoe A, Brown R, Goode M, Mongelos G (2011). Florid cemento-osseous dysplasia and a dental abscess. Dent Today.

[12] Tonioli MB, Schindler WG (2004). Treatment of a maxillary molar in a patient presenting with florid cemento-osseous dysplasia: a case report. J Endod.

[13] Cavalcante MB, Lima ALO, Brêda MA, Santos MB (2016). Florid Cemento-Osseous Dysplasia simultaneous the chronic suppurative osteomyelitis in mandible. J Craniofac Surg.

[14] Koklu HK, Çankal AD, Bozkaya S, Ergün G, Bar B (2013). Florid cemento-osseous dysplasia Report of a case documented with clinical, radiographic, biochemical and histological findings. J Clin Exp Dent.

[15] Singer SR, Mupparapu M, Rinaggio J (2005). Florid cemento-osseous dysplasia and chronic diffuse osteomyelitis: report of a simultaneous presentation and review of the literature. J Am Dent Assoc.

[16] Delai D, Bernardi A, Felippe GS, Teixeira CS, Felippe WT, Felippe MCS (2015). Florid cemento-osseous dysplasia a case of misdiagnosis. J Endod.

[17] Fenerty S, Shaw W, Verma R, Syed AB, Kuklani R, Yang J, Ali S (2017). Florid cemento-osseous dysplasia review of an uncommon fibro-osseous lesion of the jaw with important clinical implications. Skeletal Radiol.

[18] Huh JK, Shin SJ (2013). Misdiagnosis of florid cemento-osseous dysplasia leading to unnecessary root canal treatment a case report. Restor Dent Endod.

[19] Consolaro A (2014). Bisphosphonates opinion or scientific-based restrictions?. Dental Press Implantol.

